# Exploration of diagnostic biomarkers, microenvironment characteristics, and ursolic acid's therapeutic effect for benign prostate hyperplasia

**DOI:** 10.7150/ijbs.85739

**Published:** 2023-08-15

**Authors:** Yanbo Chen, Hui Xu, Huan Xu, Chong Liu, Ming Zhan, Zhong Wang, Meng Gu, Qi Chen, Bin Xu

**Affiliations:** 1 Department of Urology, Shanghai Ninth People's Hospital Affiliated to Shanghai Jiao Tong University School of Medicine, Shanghai 200011, China.; 2 Department of Emergency, Shanghai Ninth People's Hospital Affiliated to Shanghai Jiao Tong University School of Medicine, Shanghai 200011, China.

**Keywords:** diagnostic biomarkers, microenvironment characteristics, machine learning, nomogram, ursolic acid, benign prostatic hyperplasia, prostate cancer

## Abstract

Benign prostatic hyperplasia (BPH) and early-stage prostate cancer (PC) have similar symptoms, making it challenging to differentially diagnose these two conditions. The study used Weighted Gene Co-Expression Network Analysis, as well as two machine learning strategies to identify BPH-specific biomarkers based on an integrated transcriptome data from 922 samples. Eight prognostic genes (*ALCAM*, *COL6A2*, *CRISP2*, *FOXF2*, *IGF1*, *PTN*, *SCN7A*, and *UAP1*) were identified to be BPH-specific biomarkers with high accuracy and specificity. Moreover, we constructed a seven-gene diagnostic classifier to distinguish BPH from PC. The infiltrations of plasmacytoid dendritic cells and neutrophil cells showed distinct differences between BPH and non-BPH groups. Additionally, ursolic acid can reverse transcriptional features associated with the occurrence and progression of BPH. Both *in vivo* and *in vitro* experiments have confirmed that it induces apoptosis of BPH cells and inhibits cell proliferation by promoting cell cycle S-phase arrest. The diagnostic biomarkers, microenvironment characteristics, and therapeutic effect of ursolic acid explored in this study offer new diagnostic and therapeutic strategies for BPH.

## Introduction

Benign prostatic hyperplasia (BPH) has been regarded as a common disease in aging men. BPH prevalence rates ranged from 50% to 75% among males aged over 50 years to 80% among males aged over 70 years 1. The BPH leads to a physical and economic burden for patients, such as pain and suffering, treatment cost, and earning lost. For most of the patients, symptoms would significantly become worse with increasing age and time if received no treatment. Approximately 80% of men with the age over 70 years may be affected by BPH 1, 2. Since BPH growth is inexorable with aging, the economic costs of BPH treatment will continuously increase in the future 2. Recent studies have revealed the factors that affected the pathogenesis and development of BPH 3. The age, sedentary lifestyle, smoking, excessive alcohol consumption etc. have been reported as BPH-associated risk factors 4.

The definition of BPH is associated with histological enlargement in the volume of the prostate. Since the long disease course of BPH, its management has been complicated 5. The diagnosis of BPH should rely on both medical history and physical examination, including digital rectal examination and urinalysis. Other tests involved clinical symptoms, including prostate-specific antigen (PSA), serum creatinine, urine cytology, imaging, cystourethroscopy, post-void residual, and pressure-flow studies 6. The symptoms would also assist in diagnoses such as emptying and retention disorders. Several strategies have been developed to treat BPH. Transurethral resection of the prostate has been considered the gold standard for operative treatment. However, considering the suffering and pain, operation should be reserved for patients who either have failed other medical management or have complications from BPH, such as recurrent urinary tract infections, refractory urinary retention, etc 7. Although there are various medical and surgical treatment approaches, the guidelines have suggested a primary care approach 8, 9. Thus, the early diagnosis and management of BPH have become an important issue for improving the treatment benefit and reducing the pain of patients with early BPH.

The early diagnosis of BPH is challenging for the following reasons. First, until now, there is no widely recognized diagnostic marker between BPH and healthy individuals. The clinical diagnosis and treatment remained to be greatly optimized by seeking for better and more comprehensive diagnostic strategy 10, 11. Second, it lacks large sample cohort data of BPH due to its varied and mild symptoms. Thus, limited information can be found on its early clinical features. Few systematic studies have been performed on the differences among BPH, healthy prostate tissues, and prostate cancer (mainly prostate adenocarcinoma, PC) tissues, such as specific markers and immune infiltration features. Third, the differential diagnosis of BPH and PC has been difficult. BPH is often characterized by difficulties in urination and urinary retention, while early-stage prostate cancer, although lacking typical symptoms, can present clinical manifestations similar to those of benign prostatic hyperplasia, such as difficulties in urination. It is of great clinical significance to distinguish BPH from PC 11. At present, PSA has been widely used as a specific biomarker in clinical practice, but its diagnostic specificity and sensitivity are still inadequate for accurately differentiating between BPH and healthy individuals 12.

With the advances in genome and proteome technology, some innovative biomarkers have also been investigated. Researchers have tried to apply bioinformatic tools to screen significant markers for BPH. Since BPH exhibit unique immunogenic features, the immune relevant signature can also be applied for differentiating BPH, such as the immune infiltration patterns. In this study, we integrated the transcriptome data of 922 samples from Gene Expression Omnibus (GEO), Genotype-Tissue Expression (GTEx), and The Cancer Genome Atlas (TCGA), including 171 cases of healthy prostate tissues, 79 cases of BPH tissues, and 672 cases of PCA tissues. A total of 8 BPH-specific diagnostic genes were screened by the Weighted Gene Co-Expression Network Analysis (WGCNA) and two machine learning strategies. In addition, the profile of 28 kinds of immune cells specific to BPH was proposed, which preliminarily revealed the immune microenvironment characteristics of BPH. Further, the relevant bioinformatics analysis results were verified with quantitative real-time PCR and immunohistochemical detection on clinical samples from BPH, PC, and normal cases. Additionally, ursolic acid can reverse transcriptional features associated with the occurrence and progression of BPH. Both *in vivo* and *in vitro* experiments have confirmed that it induces apoptosis of BPH cells and inhibits cell proliferation by promoting cell cycle S-phase arrest. We believe that these results may provide new diagnostic and therapeutic strategies for BPH.

## Material and Methods

### Data resources and preprocessing

This study analyzed seven transcriptome data sets from GEO, GTEx, and TCGA databases, which comprised a total of 922 tissue samples, including 171 healthy prostate tissues, 79 BPH tissues, and 672 prostate cancer tissues. Detailed information on the data sets, including sample information and platform information, can be found in Table 1. To standardize the gene expression data, we applied the normalizeBetweenArrays function in the limma R package 13 to the chip-based sequencing data and used the standardized data of fragments per kilobase of exon model per million mapped fragments (FPKM) for the second-generation sequencing data. All of the data were processed by log2(x+1). To remove the batch effect of the data sets, we applied the Combat function of the SVA R package 14. After preprocessing, we included 10,376 genes in the integrated expression profile.

### Differentially expressed genes (DEGs) and functional enrichment analysis

To identify differentially expressed genes (DEGs) among the Normal, BPH, and PC groups, we applied the ebayes function in the limma package of R to the preprocessed expression profile. We set the screening threshold as an absolute value of log2Fold change > 0.585 and FDR < 0.05 for the comparison between two groups. We used a venn diagram to screen the BPH-specific DEGs that were both up or down-regulated in two comparisons: BPH vs Normal and BPH vs PC. We used the ClusterProfiler R package to investigate the GO and KEGG pathway enrichment of these DEGs. Furthermore, we conducted additional difference analyses between one group and the other two groups, and ranked all the DEGs based on their log2FoldChange. Using the ClusterProfiler R package, we analyzed the enrichment of the GSEA gene sets. Then, for both the Hallmark and KEGG gene sets, we determined specific up-regulation pathways of each group, ranked according to the Normalized Enrichment Score (NES) from high to low.

### Weighted Gene Co-Expression Network Analysis

To identify co-expressed gene modules related to phenotype, we analyzed the integrated expression profiles using the WGCNA R package 15. Firstly, we calculated the mean absolute deviation (MAD) for the expression of each gene and included the top 5000 genes in the subsequent analysis. Next, we utilized the standard WGCNA process to screen for gene co-expression modules most closely associated with the BPH phenotype.

### Machine learning

We used Support Vector Machine-Recursive Feature Elimination (SVM-RFE) and Least Absolute Shrinkage and Selector Operation (LASSO) logistic regression to further screen the feature variables in the overlapping genes of BPH-related blue modules and DEGs. For SVM-RFE, we utilized k-fold cross-validation to filter the feature variables with the e1071 R package and estimated generalization errors using different feature combinations. The feature genes with the smallest errors were selected. With LASSO logistic regression, we used the cv.glmnet function of the glmnet R package to screen the characteristic genes corresponding to lambda.min with 10-fold cross-validation.

### Immune cell infiltration analysis

For each individual sample, we implemented the estimate R package to obtain their ImmuneScore and StromalScore values, which can serve as descriptors of their immune and matrix microenvironments. Moreover, to provide a more detailed understanding of immune cell infiltration, we utilized the ssgsea method from the GSVA R package to quantitatively score the degree of infiltration of 28 distinct immune cell types - based on a background gene set from prior research 16. We also used the Pearson correlation test to assess the relationship between the eight previously identified BPH-specific diagnostic genes and immune cell infiltration within the BPH and PC groups. Additionally, we employed the boruta R package to rank the classification importance of each immune cell for both BPH and Non-BPH, followed by utilizing the pROC R package to determine the AUC value.

### qRT-PCR

The qRT-PCR experiments were conducted with the PrimeScript RT Reagent Kit and SYBR Premix Ex Taq as directed by the manufacturer's instructions. GAPDH was applied as the internal control. The primer sequences were provided in Table 2. The Ct values were obtained and analyzed with the 2^-ΔΔCt^ method.

### Immunohistochemistry

Tissue sections were deparaffinized and rehydrated, and then antigen was extracted using a 10 mM Na-Citrate buffer. The sections were treated with 0.3% H_2_O_2_ for 15 min to inhibit the endogenous peroxidase. The primary antibodies (anti-CD123 antibody, 1:50, #23797, CST and anti-CD15 antibody, 1:50, #54192, CST) were used for staining after blocking with 10% goat serum for 30 min. The quantitative analyses were utilized with Image Pro Plus software based on the mean optical density (MOD). The *t*-test was used to do differential analyses in the GraphPad Prism software.

### Integration of a diagnostic classifier to distinguish PC from BPH

To begin, we randomly divided the samples into training and validation sets in a 1:1 ratio using the createDataPartition function from the caret R package. We then employed the expression profiles of diagnostic genes and utilized the glmnet R package to construct a classifier through lasso logistic regression. Patients with a classifier score exceeding 0.5 were identified as having PC, while those with scores below 0.5 were considered as having BPH. Next, ROC analysis was conducted on both the training and testing sets. Furthermore, the accuracy of the model was evaluated by its AUC value. Finally, we utilized the rms R package to establish the diagnostic nomogram and plotted the calibration curve with the calibrate function.

### Small molecule drugs prediction

SPIED3 is an internet-based tool (http://www.spied.org.uk/cgi-bin/hgnc-spied3.cgi) that utilizes the Connectivity Map 2.0 dataset 17, which entails the profiling of 1,309 compounds on human cell lines. By inputting data regarding BPH-specific DEGs and log_2_Foldchange data, correlated expression profiles of various compounds were predicted using the Pearson correlation test. The small molecule drugs with negative correlations were then identified as potential therapeutic drugs for BPH.

### Induction and pharmacotherapy in BPH rats

Thirty castrated rats were randomly assigned to five groups (n=6): (A) control group, received PBS orally and corn oil subcutaneously; (B) BPH group, received PBS orally and testosterone propionate (TP, 3mg/kg) subcutaneously; (C) Rescinnamine group (Rescinnamine, HY-A0220, MCE, USA), received reserpine (5mg/kg) orally and TP (3mg/kg) subcutaneously; (D) Lumicolchicine group (Lumicolchicine, L474300, TRC, Canada), received Lumicolchicine (5mg/kg) orally and TP (3mg/kg) subcutaneously; (E) Ursolic acid group (Ursolic acid, HY-N0140, MCE, USA), received Ursolic acid (5mg/kg) orally and TP (3mg/kg) subcutaneously. All rats received treatment once daily for four weeks. During the experiment, the weight of each animal was measured once a week. After the final treatment and an overnight fast, all animals were anesthetized with pentobarbital (100 mg/kg, i.p.), and the entire prostate was immediately removed and weighed, and its volume was measured using a measuring cylinder. The formula for calculating the Prostate index (PI) of rats is prostate wet weight (mg) divided by body weight (g).

### HE staining

In order to evaluate the morphological changes and collagen deposition in the prostates of each group of rats, the prostate tissues were embedded in paraffin, cut into 4 µm thick slices, and stained with H&E solution (hematoxylin-eosin, Sigma MHS-16 and eosin, Sigma HT110-1-32) and Masson solution (Yeasen, China). After neutral gum sealing, the stained prostate tissues of each group were observed using a microscope (Nikon, Japan).

### Cell culture

Human benign prostatic hyperplasia cell line (BPH-1) was obtained from ATCC (USA) and cultured in high-glucose DMEM medium (Gibco, USA) containing 10% fetal bovine serum. Cell culture was performed in a constant temperature incubator at 37 ℃ with 5% CO_2_.

### CCK-8 assay

BPH-1 cells were evenly dispersed in complete culture medium and seeded into 96-well plates at a density of 1×10^4^ cells per well. After 24 hours of incubation, the cells were treated with Ursolic acid at different concentrations (0, 10, 20, 30 μmol/L) for 24 hours. DMEM culture medium and CCK-8 reagent (Vazyme, China) were mixed in a ratio of 10:1 and added to the cells, then incubated in a cell culture incubator for 2 hours. Finally, the absorbance was measured at 450 nm using an enzyme immunoassay instrument.

### Annexin V/PI double staining

Cells were seeded at a density of 1×10^5^ cells per well in 6-well plates. The experiment was divided into a blank group and a Ursolic acid group. After 24 hours of drug administration, the cells were digested with trypsin and washed twice with PBS. The cells were then centrifuged at 3000 rpm for 5 minutes, and the supernatant was removed. 500 µL of cell suspension containing 1×Annexin V-FITC binding solution was prepared. To the cell suspension, 5 µL of Annexin V-FITC staining solution and 5 µL of PI staining solution were added. The suspension was placed in a dark room at room temperature for 15 minutes, and then analyzed using a flow cytometer. Finally, the results were analyzed using Flow Jo software.

### Cell cycle detection

Flow cytometry was used to detect the effects of Ursolic acid treatment on the cell cycle. The experiment was divided into a blank group and an Ursolic acid group. After 24 hours of drug administration, the cells were fixed overnight in 70% anhydrous ethanol, then stained with 50 mg/mL PI at 4°C for 30 minutes. Finally, the cell cycle was assessed using a flow cytometer. The cell cycle was represented by G0-G1, S, and G2-M.

### Western Blot

The experiment was divided into a blank group and an Ursolic acid group. 1×10^6^ BPH-1 cells were suspended in 200 μL of RIPA buffer. The cells were then lysed and the total protein was extracted from the cells. The protein concentration was measured using the BCA protein assay. The protein sample was loaded onto a 10% SDS-PAGE gel, and the protein sample was separated by electrophoresis. The separated proteins were then transferred onto a PVDF membrane. The membrane was blocked with 5% skim milk at room temperature for 60 minutes. Then, the membrane was incubated with corresponding primary antibodies overnight at 4℃. The primary antibodies included: anti-Bcl-2 (1:1000, ab32124, abcam), anti-Bax (1:1000, ab32503, abcam), anti-Caspase3 (1:1000, ab32351, abcam), and anti-GAPDH (1:1000, ab8245, abcam). The membrane was washed three times with TBST for 5 minutes each time. Then, it was incubated with HRP-conjugated goat anti-rabbit or anti-mouse antibodies (1:2000) on a shaker for 1 hour. The membrane was washed three times with TBST for 5 minutes each time, and then developed using an ECL detection kit. GAPDH was used as an internal control, and the protein band gray value was analyzed using Image J.

### Statistical analysis

Bioinformatics data analysis was performed using R version 4.1.3. Experimental data was presented as mean ± standard deviation (SD) and analyzed using GraphPad Prism 8.0.1 (GraphPad, San Diego, CA, United States). One-way or two-way ANOVA was used to evaluate the significance of multiple groups, and Tukey or Dunn's multiple comparison tests were used. A p-value < 0.05 was considered statistically significant.

## Results

### Identification of BPH-specific DEGs and functional enrichment analysis

**Figure 1:**
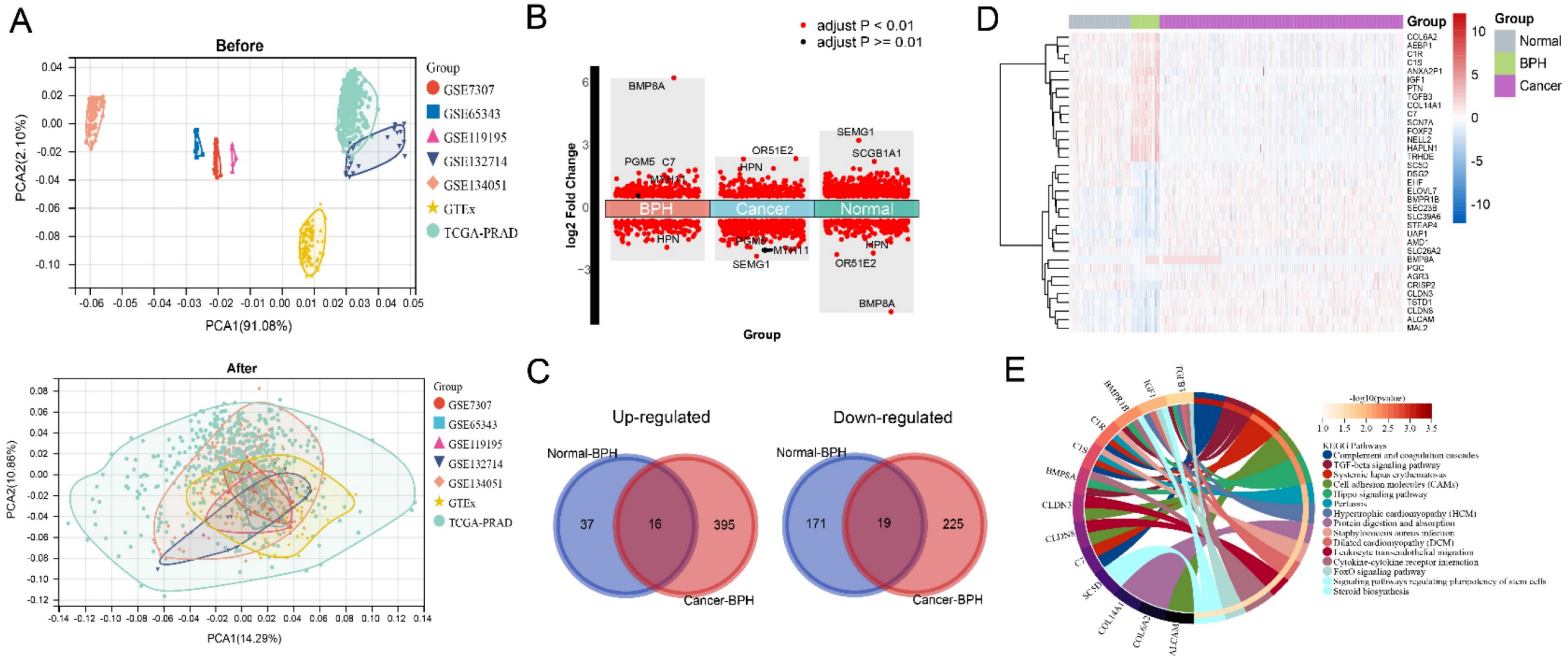
**Identification of BPH-specific DEGs.** (A) The PCA before and after removing batch effect. (B) The distribution of DEGs and Top 5 characteristic genes in BPH, PC, and Normal groups. (C) Wayne diagram of BPH-specific 16 up-regulated genes and 19 down-regulated genes. (D) The heatmap of the expression profile of DEGs in BPH, PC, and Normal groups. (E) The enrichment analysis of the KEGG pathway for the 35 BPH-specific DEGs.

**Figure 2:**
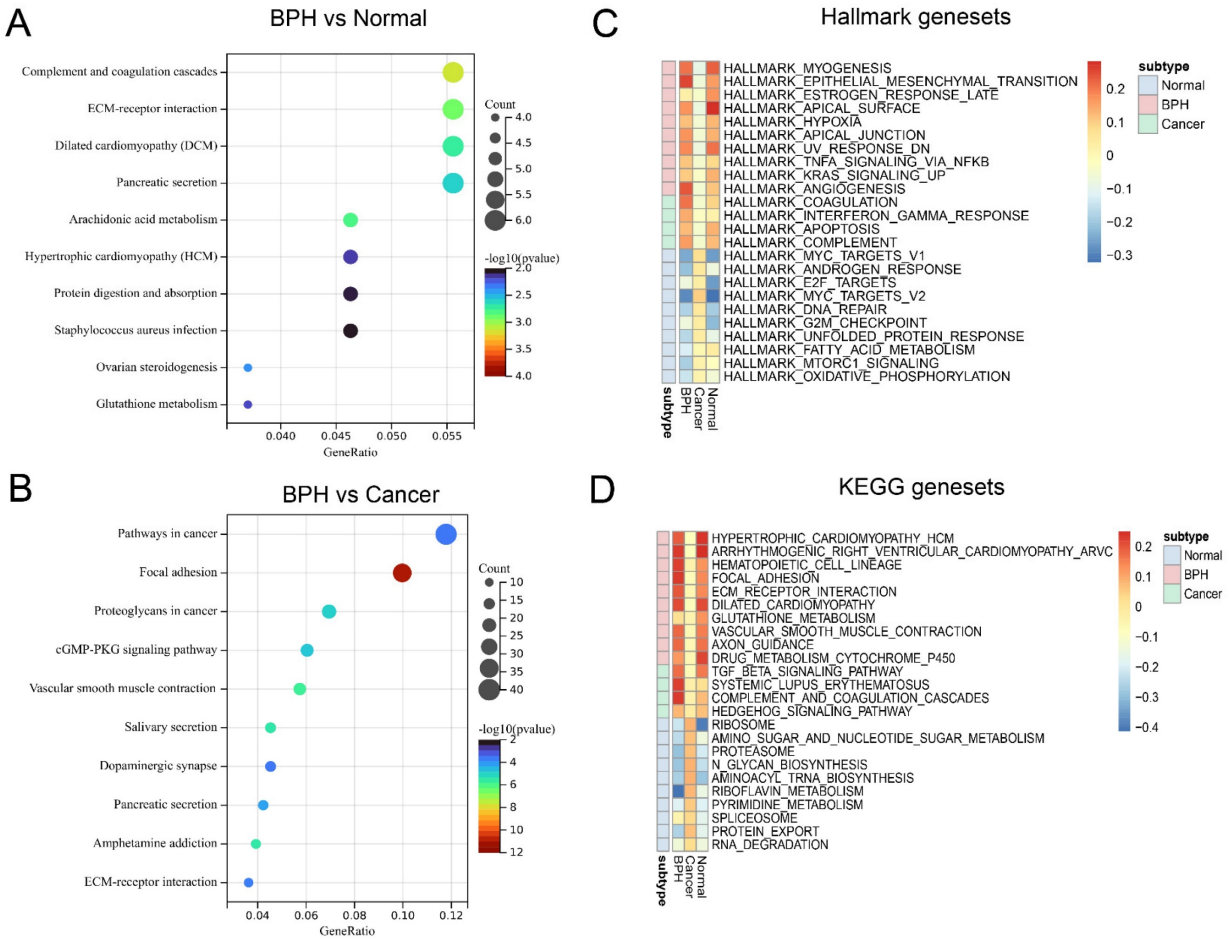
** Function enrichment analysis of BPH-specific DEGs.** (A) The function enrichment analysis of DEGs between BPH and Normal group. (B) The function enrichment analysis of DEGs between BPH and PC groups. (C) The Hallmark gene sets of BPH, PC, and Normal groups. (D) The KEGG gene sets of BPH, PC, and Normal groups.

The workflow of this study is shown in Figure S1. The results of principal component analysis (PCA) suggest that batch effects exist in the 7 datasets. Once the batch effect is removed, the data distribution is adjusted to an acceptable range, enabling the comparability of data (Figure 1A). DEGs and characteristic genes are screened among the Normal, BPH, and PC groups after conducting the difference test. The distribution of DEGs and the top 5 characteristic genes are shown (Figure 1B), and after using the venn diagram, a total of 16 up-regulated genes and 19 down-regulated genes specific to BPH are obtained (Figure 1C). The expression profile of these DEGs is presented in a heatmap (Figure 1D). Statistical results of DEGs in each group are provided in Supplementary File S1. The KEGG pathway of enrichment analysis reveals that the 35 DEGs are mainly enriched in pathways associated with the immune system and matrix microenvironment, such as the Complement and coagulation cascades, TGF-beta signaling pathway, Cell adhesion molecules (CAMs), Hippo signaling pathway, Leukocyte transendothelial migration, and Cytokine-cytokine receptor interaction (Figure 1E). The DEGs between the BPH and Normal groups significantly enriched in functions such as Complement and coagulation cascades and ECM-receptor interaction (Figure 2A). The DEGs between the BPH and PC groups are significantly enriched in Pathways in cancer, Focal adhesion, and Proteoglycans in cancer (Figure 2B), which suggest that there may be crucial differences in the matrix microenvironment. By conducting GSEA analysis, Hallmark or KEGG pathways specifically upregulated in each group were screened. It was found that similar expression patterns exist between the BPH and Normal groups, and these patterns differ greatly from PC. Compared with the two other groups, BPH significantly up-regulates the pathways of Epithelial-mesenchymal transition, Angiogenesis, Coagulation, Hematopoietic cell lineage, Focal adhesion, ECM-receptor interaction, Complement and coagulation cascades (Figure 2C and 2D).

### Screening BPH-related co-expression gene modules by WGCNA

**Figure 3:**
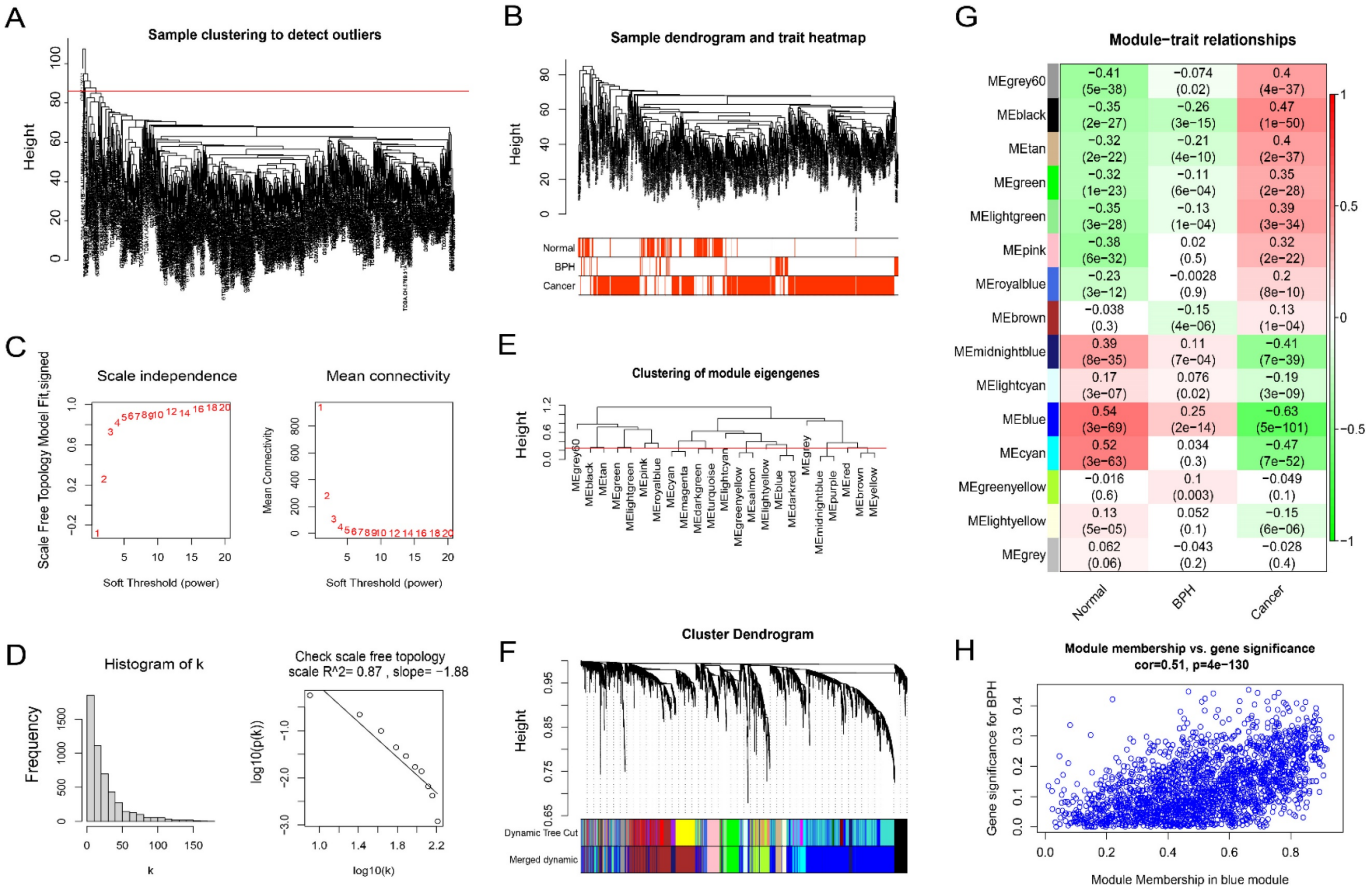
** BPH-related co-expression gene modules screened by WGCNA.** (A) Sample clustering to detect outliers. (B) The cluster tree and phenotype information of the outliers for BPH, PC, and Normal groups. (C) The scale-free topology model fit, which shows the change trends of Scale Independence and Mean Connectivity with the Soft Threshold (power). (D) The relationship with Mean connectivity. (E) The clustering of module eigengenes. (F) The sample cluster tree after merging similar modules with a distance of less than 0.25, which contains 15 modules. (G) Pearson correlation of module and phenotype for BPH, PC, and Normal groups, in which the blue module has the greatest correlation with BPH. (H) The Module membership and gene significance in the blue module.

Firstly, the outliers are removed by the goodSamplesGenes function included in the WGCNA R package (Figure 3A). The cluster tree and phenotype information of the outliers are displayed (Figure 3B). Then, the power is determined as 5 through the powerEstimate function, and the scale-free network is established. The change trends of Scale Independence and Mean Connectivity with the Soft Threshold (power) are shown (Figure 3C). The construction of the final scale-free network is shown (Figure 3D). The left panel shows a histogram of network connectivity. The right panel shows a log-log plot of the same histogram. The high R^2^ value of 0.87 shows approximate scale-free topology (Figure 3D). Then, similar modules whose distance is less than 0.25 are merged and labeled with different colors (Figure 3E). The merged sample cluster tree is shown (Figure 3F), which contains 15 modules. Pearson correlation test of module and phenotype shows that, the blue module has the greatest correlation with BPH (coefficient = 0.25, *P* = 2e^-14^), and the greatest negative correlation with PC (coefficient = -0.63, *P* = 5e^-101^), and the highest positive correlation with the Normal group (coefficient = 0.54, *P =* 3e^-69^). It suggests that the genes in this module are potential diagnostic genes to distinguish BPH or Normal samples from PC (Figure 3G). The Module membership and gene significance in the blue module also show a high correlation (coefficient = 0.51, *P* = 4e^-130^) (Figure 3H). The correlation results between genes and phenotypes of each module are provided in Supplementary File S2.

### Screening BPH-specific diagnostic genes with machine learning

**Figure 4:**
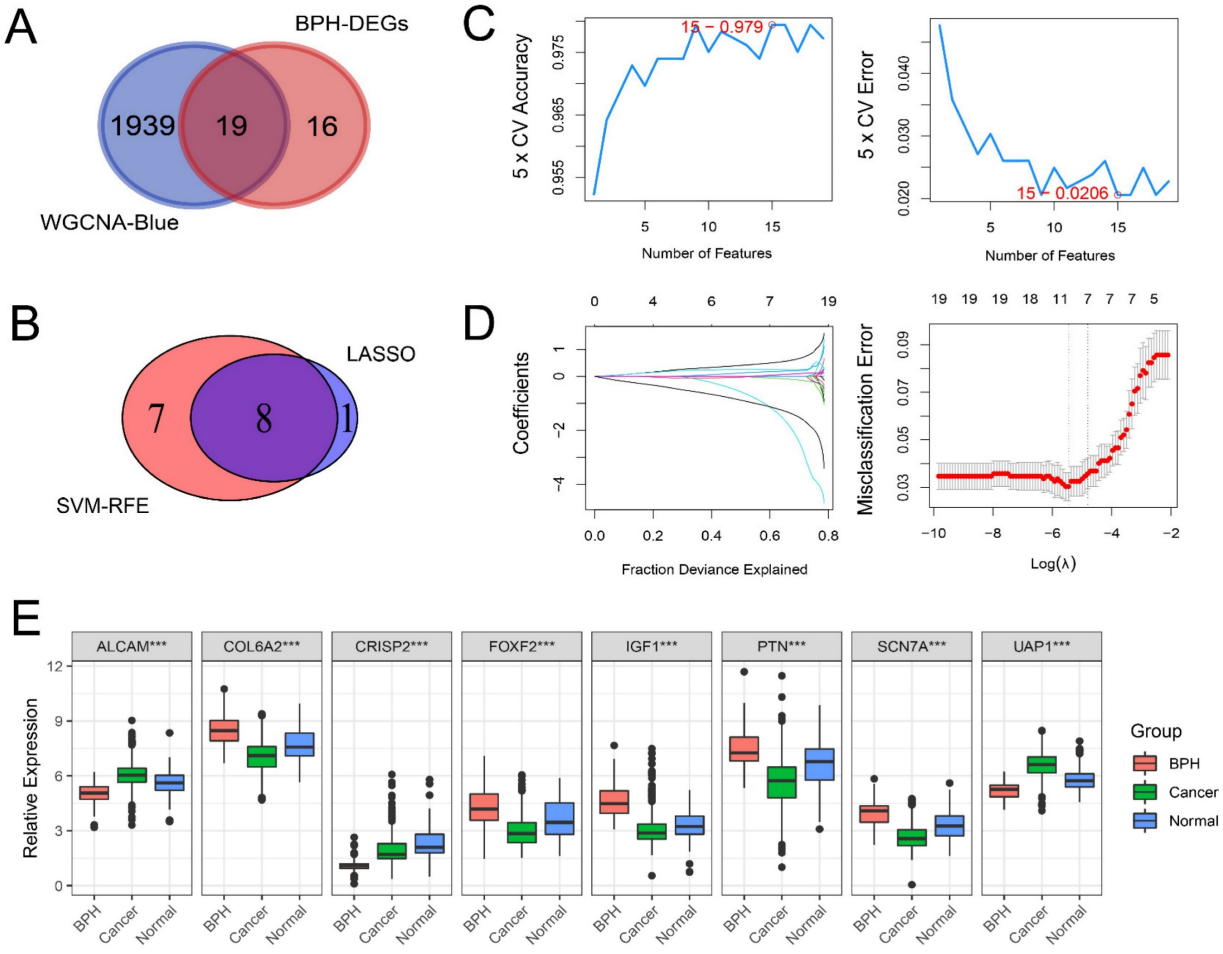
** BPH-specific diagnostic genes are screened with machine learning.** (A) The 19 overlapping genes between genes in the blue module of WGCNA and BPH DEGs. (B) The 8 diagnostic genes were identified by SVM-RFE and LASSO methods (*ALCAM*, *COL6A2*, *CRISP2*, *FOXF2*, *IGF1*, *PTN*, *SCN7A*, and* UAP1*). (C) The different variable combinations with corresponding accuracy and errors. (D) The feature genes corresponding to the minimum value of lambda in LASSO logistic regression. (E) The expression box diagram of 8 identified genes in BPH, PC, and Normal groups.

**Figure 5:**
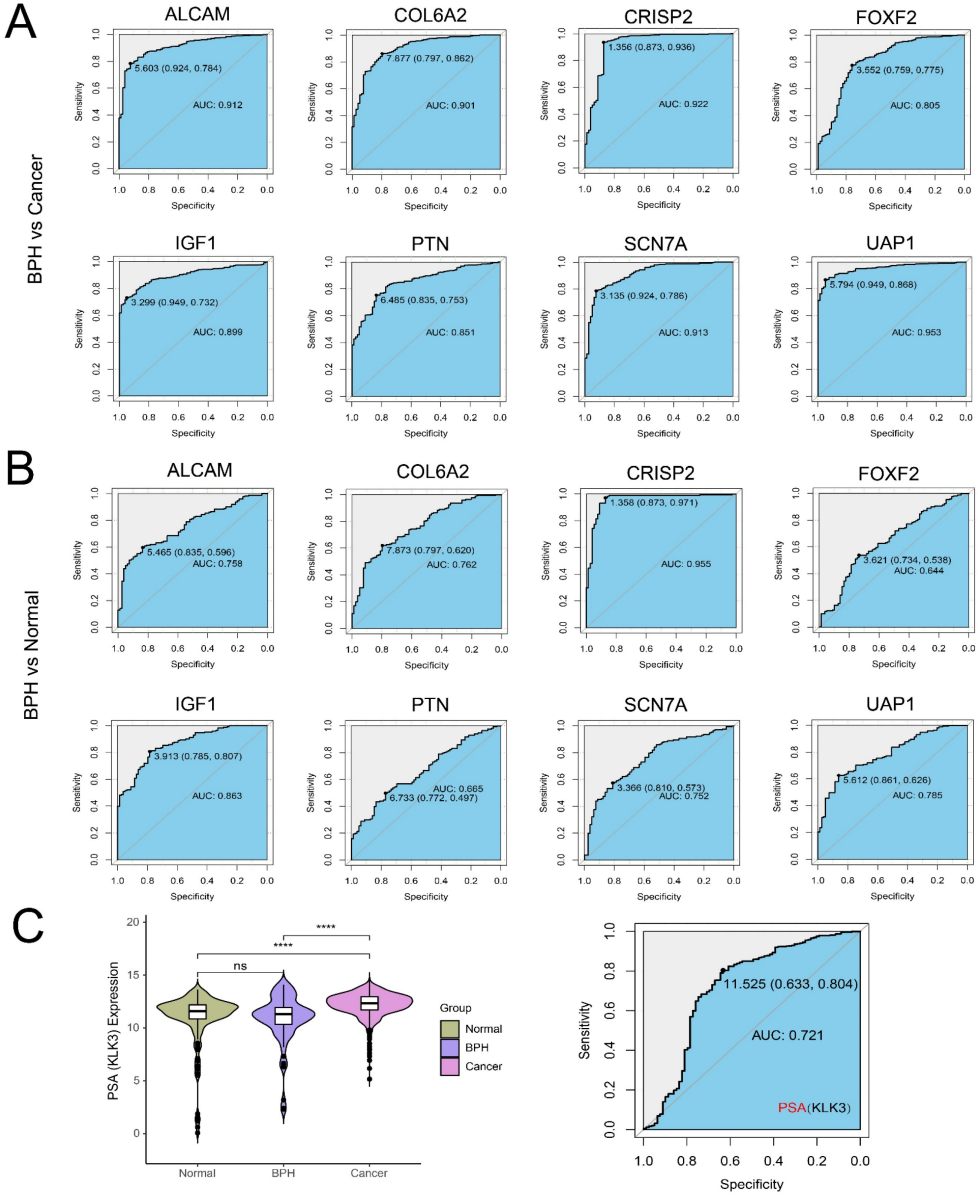
** The ROC curves of 8 identified diagnostic genes (*ALCAM*, *COL6A2*, *CRISP2*, *FOXF2*, *IGF1*, *PTN*, *SCN7A*, and* UAP1*).** (A) Comparison between BPH and PC groups; (B) Comparison between BPH and Normal groups. (C) The difference of KLK3 gene encoding PSA protein among BPH, PC, and Normal groups (Left). The ROC curves of KLK3 between BPH and PC groups (Right).

There are 19 overlapping genes between BPH DEGs and genes in the blue module, which enter the subsequent SVM-RFE and LASSO logistic regression joint screening (Figure 4A). After screening by two different machine learning methods, 8 robust diagnostic genes are identified (Figure 4B). Specifically, for SVM-RFE, we use k-fold cross-Validation to screen feature variables and estimate generalization errors using different feature combinations to screen 15 features with the smallest errors as feature genes. The different variable combinations with corresponding accuracy and errors are shown (Figure 4C). For LASSO logistic regression, we use 10-fold cross-validation to screen the feature genes corresponding to the minimum value of lambda (Figure 4D) and plot the expression box diagram (Figure 4E) of 8 genes in three groups. Then, the ROC curves are plotted (Figure 5A and 5B). The diagnostic specificity and sensitivity of each gene are evaluated by AUC value of ROC curve. In addition, the optimal cutoff expression value is calculated (Table 3). The results show that the 8 genes exhibit high accuracy not only in distinguishing BPH from PC, but also in differentiating BPH from Normal samples. In addition, we also analyze the differences of *the KLK3* gene encoding PSA protein among the three groups. The results show that *KLK3* mRNA is significantly higher in PC groups, but there is no difference between BPH and Normal groups. Further, the AUC value for distinguishing BPH from PC is only 0.721, which is lower than those of 8 screened genes (Figure 5C).

### Analysis of immune cell infiltration characteristics

**Figure 6:**
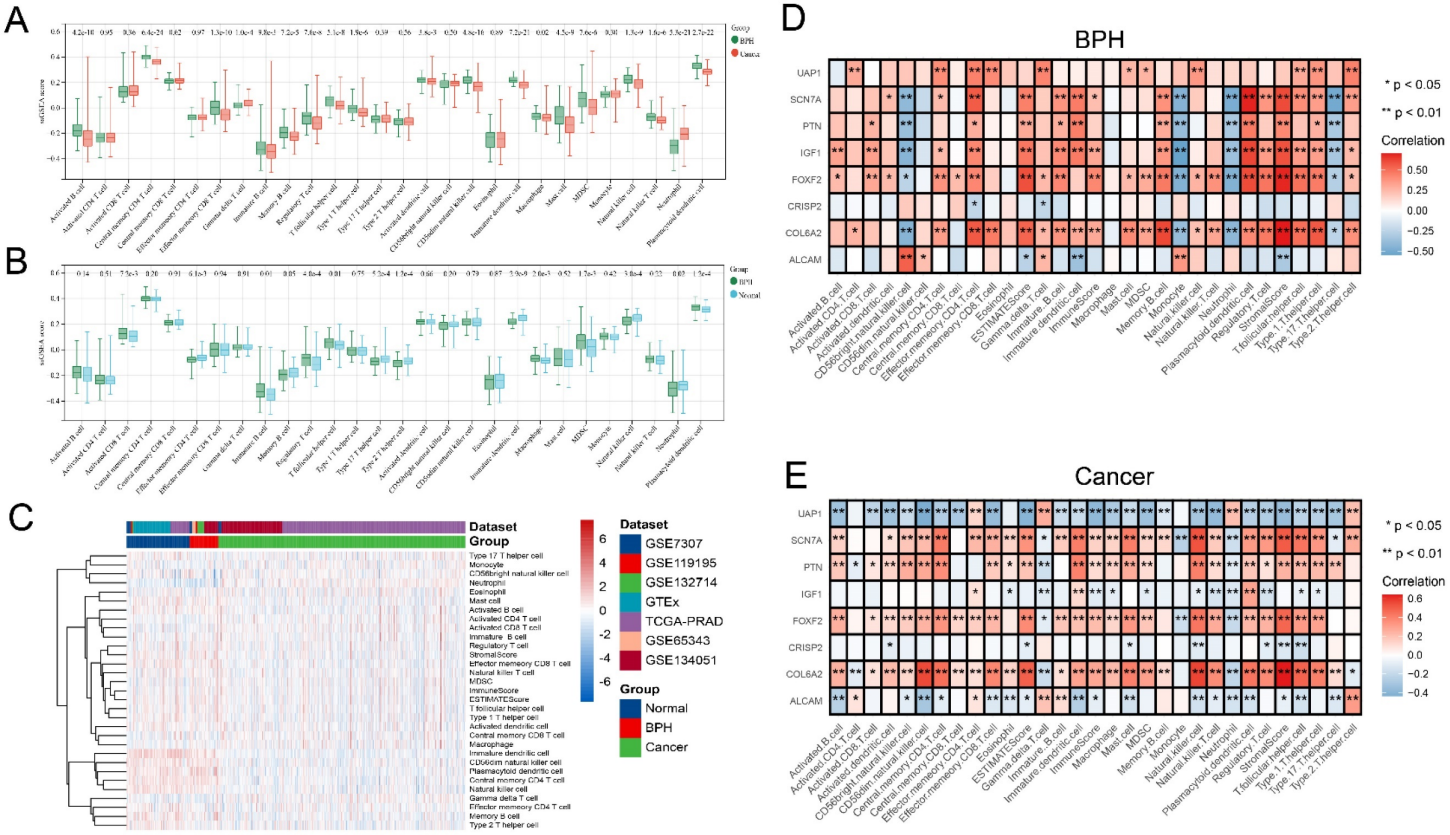
** Analysis of immune cell infiltration characteristics.** (A) The difference of 28 kinds of immune cell infiltration between BPH and PC groups. (B) The difference of 28 kinds of immune cell infiltration between BPH and Normal groups. (C) The heatmap of 28 kinds of immune cell infiltration among three groups. Pearson correlation between 8 identified diagnostic genes and 28 immune cell infiltration in (D) BPH group and (E) PC group.

**Figure 7:**
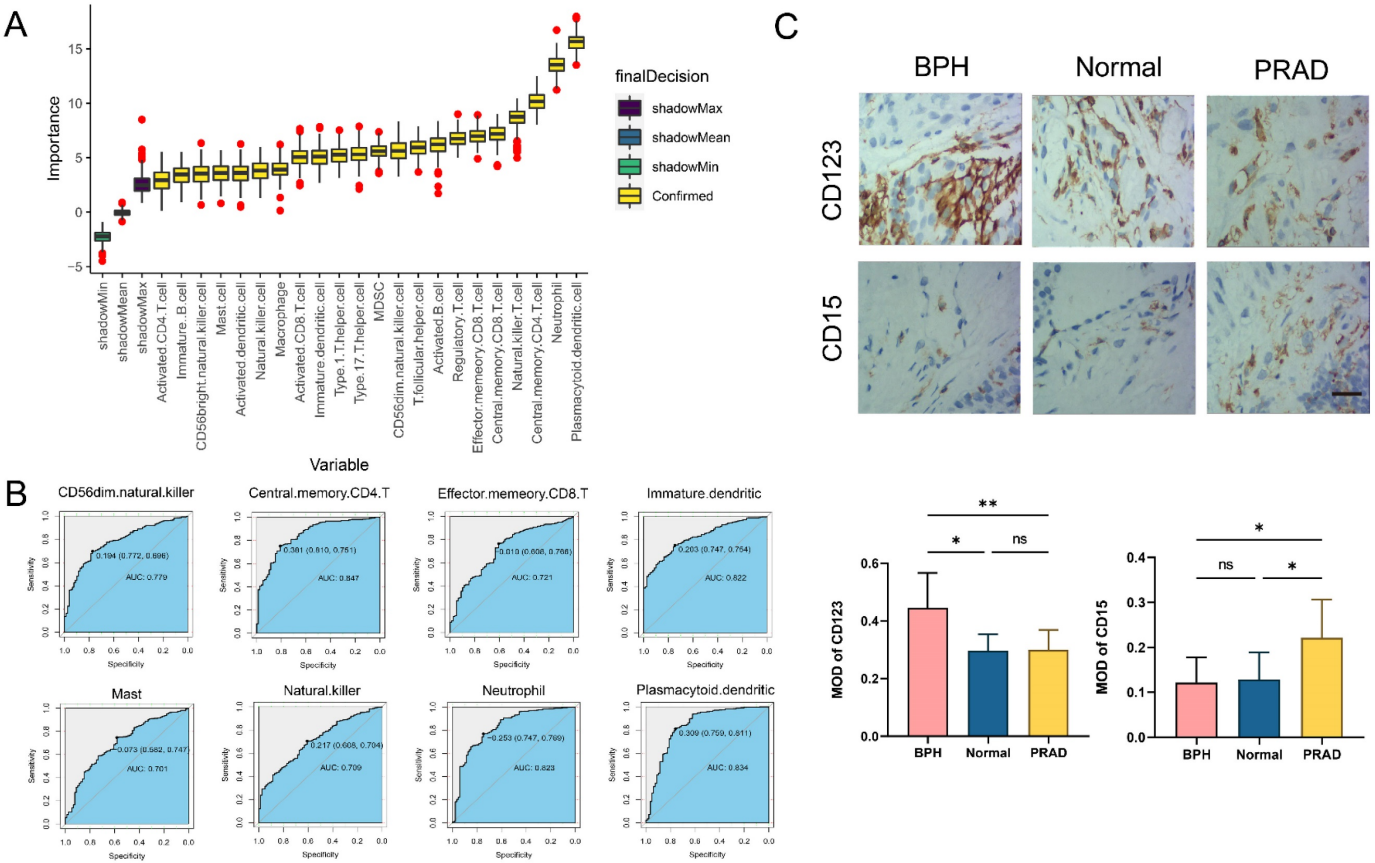
** The immune cell infiltration for distinguishing BPH from Non-BPH groups.** (A) The importance of 28 kinds of immune cell infiltration for distinguishing BPH from Non-BPH. (B) The ROC curves of 8 kinds of immune cells infiltrating with the AUC value exceeding 0.7. (C) The IHC images and MOD of CD123 and CD15 in clinical samples classified into BPH, PC and Normal groups. Statistical data were presented as Mean ± SD, and statistical significance was determined using One-way ANOVA test. "ns" indicates no significant difference, "*" indicates *P* < 0.05, and "**" indicates *P* < 0.01.

The difference analysis of 28 kinds of immune cell infiltration is performed among BPH, PC, and Normal groups. The results show that the infiltration degree of 17 kinds of immune cells in the BPH group is significantly higher than that in the PC group, while only Gamma delta T cell and Neutrophil are decreased. It is consisting of the characteristics of PC as a cold tumor (Figure 6A). There is also a big difference in immune cell infiltration between the BPH group and the Normal group. In the BPH group, the infiltration of 7 immune cells is up-regulated, while the infiltration of the other 7 immune cells is down-regulated (Figure 6B). According to the heatmap (Figure 6C), the immune infiltration in the PC group is generally low, but it is more active in BPH and Normal groups. Compared with the other two groups, the immune cells in the BPH group have differences with the same trend as follows: 6 types of immune cell infiltration are up-regulated (Immature B cell, Regulatory T cell, T follicular helper cell, Macrophage, MDSC, and Plasmacytoid dendritic cell) and 1 immune cell infiltration is down-regulated (Neutrophil). There are similarities and differences in the patterns of 8 diagnostic genes and immune cell infiltration between BPH and PC groups. The patterns of *FOXF2*, *PTN*, *SCN7A*, and *COL6A2* in BPH and PC groups are similar, which are positively correlated with the infiltration of most immune cells. *UAP1* is interesting, which shows a positive correlation in the BPH group, while a negative correlation in the PC group (Figure 6D-6E). Particularly, we note that *CRISP2* has the lowest correlation with immune cell infiltration, and there is almost no correlation result in the BPH group, suggesting that *CRISP2* may be a diagnostic marker independent of the heterogeneity of immune cell infiltration. A previous study reported that there was no correlation between the expression of *CRISP2* and androgen level 18. In previous ROC results, *CRISP2* also shows the highest AUC value, suggesting that* CRISP2* may be the diagnostic gene with the greatest potential among the 8 identified genes. The results of Boruta show that plasmacytoid dendritic cell (pDC) and Neutrophil are the most important in distinguishing BPH from Non-BPH (Figure 7A), and the AUC values of the ROC curve are 0.834 and 0.823 (Figure 7B). For differentiating BPH, there are 8 kinds of immune cells infiltrating with the AUC value exceeding 0.7 (Figure 7B). A total of 6 cases of normal prostate, 10 cases of BPH, and 8 cases of PC tissues are collected from Affiliated Ninth People's Hospital of Shanghai Jiao Tong University School of Medicine. The IHC results validated that the MOD value of CD123 (marker of pDC) in the BPH group is the highest, which is significantly higher than that in the other two groups, while the expression of CD15 (marker of Neutrophil) is very weak, which is significantly lower than that in PC group (Figure 7C). Interestingly, in a previously published retrospective clinical study, the neutrophil infiltration in PC patients was also found to be significantly higher compared to BPH patients 19, further corroborating our findings. These results preliminarily screened the potential value of key immune cell infiltration in the diagnosis of BPH.

### Validation the expressions of BPH-specific diagnostic genes in clinical tissues and efficacy prediction of small molecular compounds in BPH

**Figure 8:**
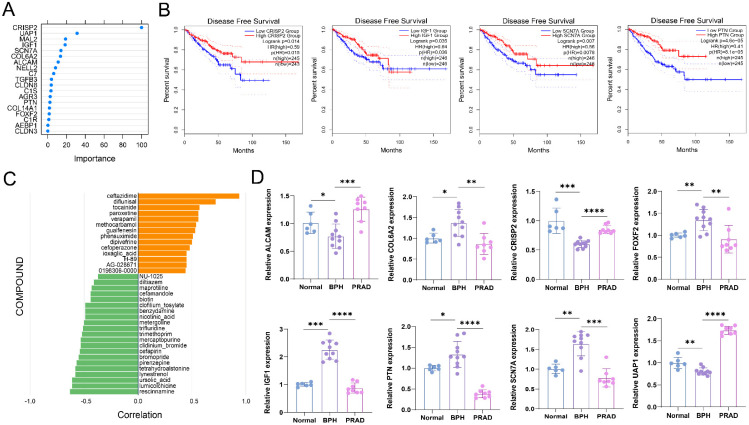
** Validation the expressions of BPH-specific diagnostic genes in clinical tissues and efficacy prediction of small molecular compounds in BPH.** (A) Random forest analysis of 19 BPH-specific diagnostic genes. (B) The expression of 8 diagnostic genes in clinical samples is classified into BPH, PC, and Normal groups, which is determined with qRT-PCR. (C) Pearson correlation between the expression profiles of DEGs and cells treated with different drugs was analyzed with the SPIED3 tool.

The results of random forest analysis show that *CRIPSP2* and *UAP1* are most important in the diagnosis of BPH (Figure 8A). The relative mRNA expression levels of 8 diagnostic marker genes are detected by qRT-PCR. The statistical results show that all 8 genes have specific differential expression in the BPH group (Figure 8B). The results are consistent with the results of bioinformatics analysis. At the same time, we find that *CRISP2*,* IGF1*,* SCN7A,* and *PTN* are prognostic markers of PC, and their high expression levels indicate a significantly better survival prognosis of DFS (Figure S3), suggesting that they are potential tumor suppressor genes. Pearson correlation between the expression profiles of DEGs and cells treated with different drugs is analyzed with the SPIED3 tool. The results show that rescinnamine, lumicolchicine, ursoloc acid, lynestrenol, etc. may be potential small molecular compounds for treating BPH (Figure 8C).

### Exploration of a diagnostic nomogram to distinguish PC from BPH

**Figure 9:**
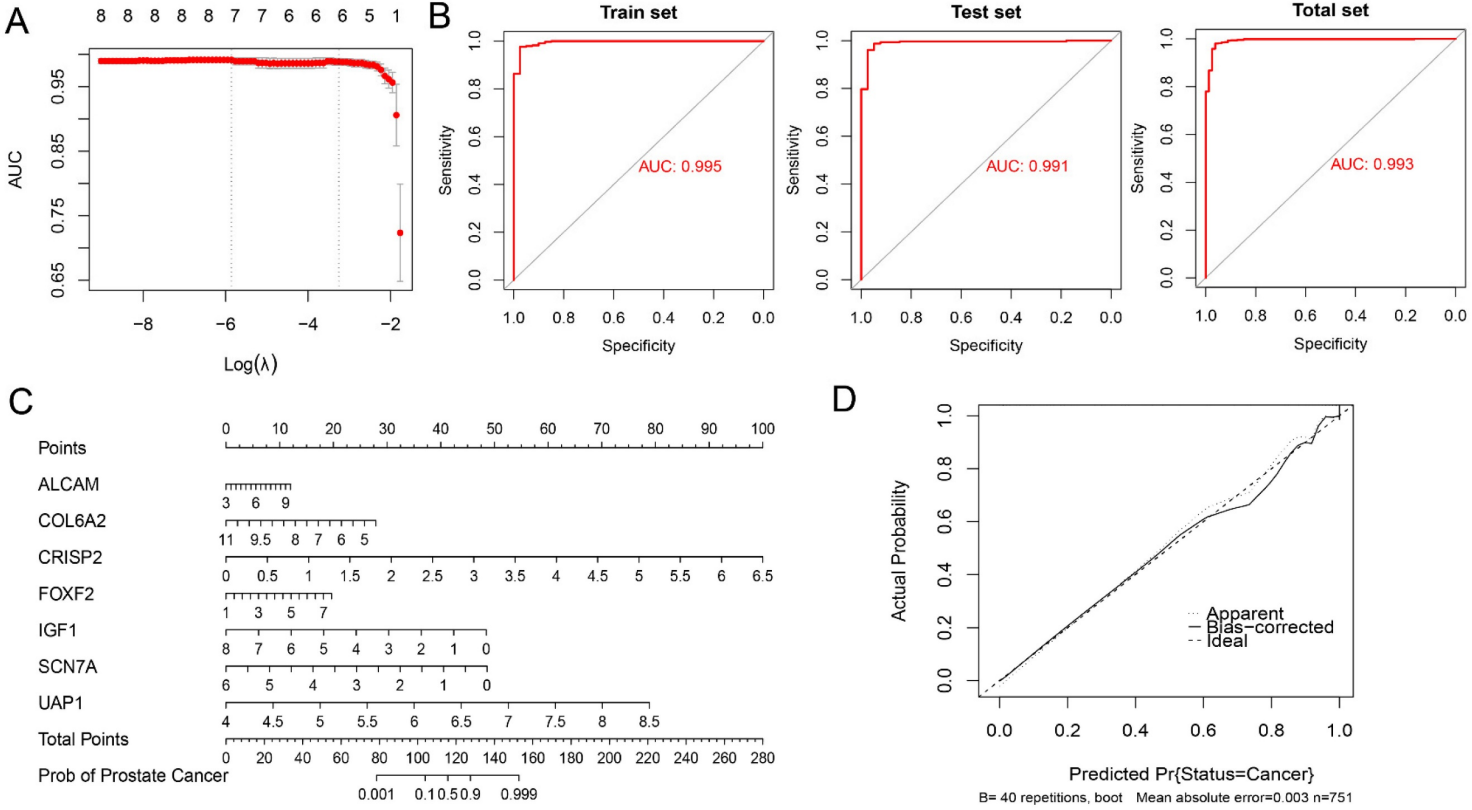
** Exploration of a diagnostic nomogram to distinguish PC from BPH.** (A) The trend of AUC value with Log(λ) in the Lasso logistic regression.** (**B**)** ROC curves of the training, testing and total sets. (C) The diagnostic nomogram to distinguish PC from BPH. (D) The calibration curve of the nomogram.

BPH and PC patients included in this study were randomly divided into the training set and testing set in a 1:1 ratio. A seven-gene logistic regression classifier with highest AUC value was explored in the training set (Figure 9A). The formula of the classifier was ALCAM*1.495 + COL6A2*(-3.312) + CRISP2*4.987 + FOXF2*1.555 + IGF1*(-3.083) + SCN7A*(-4.301) + UAP1*(13.091) - 21.653. Patients with a classifier score greater than 0.5 were diagnosed with PC. The AUC values reached 0.995, 0.991, and 0.993 in the training set, testing set, and total set (Figure 9B). A diagnostic nomogram was constructed in the total set for the potential clinical application (Figure 9C). The calibration curve showed that the result was very close to that in the ideal condition (Figure 9D).

### Ursolic acid exerts its therapeutic effect on BPH by inducing apoptosis of BPH-1 cells and affecting the cell cycle

**Figure 10:**
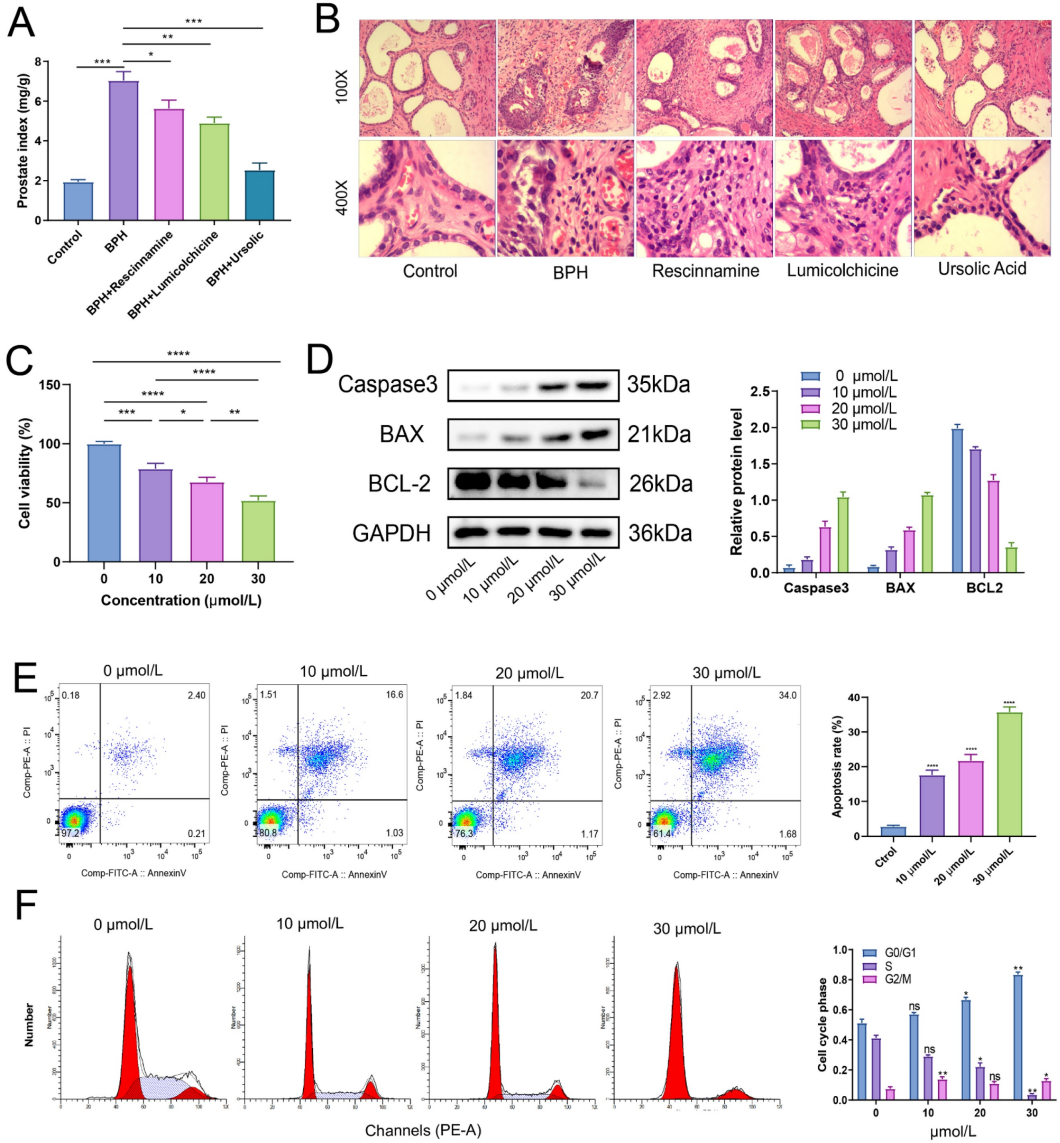
** The therapeutic effect of ursolic acid on BPH.** (A) Comparison of prostate index among different drug treatment groups. (B) Comparison of pathological morphology of prostate tissue among various treatment groups by HE staining. (C) CCK-8 experiment to detect cell viability of BPH-1 cells at different concentrations of ursolic acid. (D) Changes in protein expression of apoptosis-related proteins Caspase3, Bax and anti-apoptotic protein Bcl-2 after ursolic acid treatment of BPH-1 cells. (E) Flow cytometry to confirm the promoting effect of ursolic acid on BPH-1 cell apoptosis. (F) Flow cytometry to delineate the effect of ursolic acid on BPH-1 cell cycle. ns: not significant, *P < 0.05, **P < 0.01, ***P < 0.001, ****P < 0.0001.

After treatment for 4 weeks, the weight of each group of rats was recorded, and the wet weight and volume of the prostate tissue of each group of rats were measured. The results of the prostate index showed that all the three drugs had a good improvement effect on the prostate index of BPH rats, with the best therapeutic effect observed in the ursolic acid group (Figure 10A). Ursolic acid treatment led to significant improvement in the pathological status of the BPH prostate, with the HE staining showing an overall regular prostate glandular lumen, mostly clear glandular arrangement, single-layer columnar epithelial cells of acini, no obvious proliferation in the stromal tissue, and no obvious inflammatory cell infiltration (Figure 10B). To further clarify the effects of ursolic acid on the occurrence and development of BPH, we used ursolic acid to treat prostate hyperplasia cells (BPH-1) to evaluate its effects on BPH-1 cell activity. The CCK-8 experiment results showed that ursolic acid inhibited the viability of BPH-1 cells in a concentration limitation-dependent manner (Figure 10C). After treating cells with different concentrations of ursolic acid, the expression of apoptosis-related proteins Caspase3 and Bax gradually increased, while the expression of anti-apoptotic protein Bcl-2 gradually decreased, as detected by WB experiments (Figure 10D). Furthermore, flow cytometry detected significant induction of BPH-1 cell apoptosis (Figure 10E) and cycle arrest (mainly in the S phase) (Figure 10F) by ursolic acid treatment, which was concentration-dependent. These results suggest that ursolic acid may play a therapeutic role in BPH by inhibiting BPH-1 proliferation and inducing apoptosis through cell cycle arrest.

## Discussion

In clinical practices, BPH has become a common disease in aging men. The early evaluation of BPH has become important, which will benefit the management and treatment of BPH. In addition, the differential diagnosis of BPH and PC is significant for early treatment and primary care. Although there are some markers for the diagnosis of PC, it still lacks specific biomarkers for BPH. With the advances in genome and proteome technology, some innovative biomarkers have also been involved. The first is some biochemical indicators in the blood. The platelet to lymphocyte ratio inflammation marker has been evaluated in PC and BPH. Statistically significant differences were observed in the mean platelet lymphocyte ratio (PLR) values only if the PSA level was 10 ng/mL and above in the BPH and prostate cancer groups 20. The oxidative stress in BPH patients has been assessed for evaluating the effects of the operation. The results suggested that the oxidative stress can be better reflected by blood level of 8-hydroxy-2'-deoxyguanosine/deoxyguanosine level compared to that of the malondialdehyde, and surgical operation attenuated the oxidative stress in the late postoperative period in BPH patients 21. The combination of GC-MS based metabolomics and machine learning revealed that three metabolites could be promising indicators for distinguishing prostate cancer from BPH, which were L-serine, myo-inositol, and decanoic acid 22. The second is the genetic biomarkers, such as microRNA(miRNA). The miRNA can be applied as another kind of biomarker independent of PSA, Gleason score, or TNM status 23. The results of microarray revealed 7 deregulated miRNAs in PC patients in contrast to BPH patients. The hsa-miR-221-5p and hsa-miR-708-3p were experimentally verified to be down-regulated in prostate cancer compared to BPH. In particular, the expression ratio of urinary miR-H9 to miR-3659 can be applied for discriminating prostate cancer from BPH, particularly for patients with PSA level in gray zone 24. The third is the functional protein. Heat shock proteins (HSPs), chaperone proteins, function in maintaining cell homeostasis. HSPs made cytoprotective effects and affected the survival of cancerous cells. Chaperones were played an indispensable role in tumor progression. Therefore, HSPs have been considered targets for differentiating BPH and malignant prostate cancer 25. The inflammation-related biomarkers have attracted the attention of researchers. The inflammation has played role in the development of BPH 26. Inflammation was associated with the pathogenesis, symptoms, and progression of BPH 27. Several early candidates have been currently assessed, such as serum malondialdehyde (MDA), serum C-reactive protein (CRP), cytokines and chemokines (such as IL-8), and so on. The production of inflammatory cytokines can be derived from the infiltration cells. However, until now, few studies have been performed on seeking the characteristic genes of BPH.

A total of 8 BPH-specific genes have been screened in our study, including *ALCAM*, *COL6A2*,* CRISP2*, *FOXF2*, *IGF1*, *PTN*, *SCN7A,* and *UAP1*. Some previous studies have reported their effects on the pathology of some diseases, which mainly focused on prostate cancer. *ALCAM* has been reported to link with the progression of various cancers. One previous study has highlighted the potential of* ALCAM* as a biomarker for prostate cancer progression. Serum levels of ALCAM have shown promise as a prognostic indicator in prostate cancer, while increased tissue levels of ALCAM have been associated with a more aggressive cellular phenotype and metastasis 28. In addition to the marker, *ALCAM* is also a functional regulator of prostate cancer progression in response to TGF-β signaling 29. In a bioinformatic analysis, *ALCAM* has been integrated in the cellular senescence-related gene prognostic index to predict metastasis and radioresistance in prostate cancer 30. Our study first reported the association between *ALCAM* and BPH, and it suggested that *ALCAM* may be a shared characteristic gene of both BPH and PC. *COL6A2* encodes collagen type VI‑α, a beaded filament collagen found in most connective tissues. Collagen is the scaffold of the tumor microenvironment, regulating extracellular matrix remodeling to promote tumor infiltration, angiogenesis, invasion, and migration. *COL6A2* may act as classical collagens by forming a physical barrier to inhibit bladder cancer growth and invasion 31. Another study revealed the association between pancreatic cancer for SNPs at *COL6A2* (21q22.3) 32. *CRISP2* encodes cysteine-rich secretory protein 2. CRISPs are mainly found in the mammalian male reproductive tract and function at different stages of fertilization 33. *CRISP2* genetic defects in sperm from humans and mice result in male infertility 34. One recent study has reported that prostate secretory protein 94 inhibited sterol binding and export of *CRISP2* in a calcium-dependent manner, which affected the prostate physiology and progression of prostate cancer 35. Our results revealed that* CRISP2* can be a characteristic gene specific to BPH, since the AUC values for distinguishing BPH from normal, cancer, and non-BPH all exceeded 0.9, indicating good predicting performance. *FOXF2* encodes Forkhead box 2, which is a member of the large family of forkhead transcription factors. The expression profile of *FOXF2* suggested a role in epithelial to mesenchymal transition, which functioned in both benign and malignant outgrowths 36. The decreased expression of *FOXF2* has been reported in prostate cancer 37. Several studies have reported the association between *FOXF2* and prostate cancer, while our study first revealed that *FOXF2* can also be a significant marker for BPH. *IGF-1* encodes insulin-like growth factor 1, which is an anabolic peptide hormone that has a role in stimulating the growth of cells and tissues, including muscles and bone. A multiethnic cohort study demonstrated that *IGF1* also played a role in prostate development and carcinogenesis, while the inherited variation of *IGF1* may affect the risk of prostate cancer 38. Obesity has been reported as a risk factor for BPH. The *IGF1* axes could play a pathophysiological role in affecting prostate cell function 39. Another study proposed an innovative perspective, that the intestinal bacteria, acting through short-chain fatty acids, regulate systemic and local prostate IGF1 in the host, which can promote the proliferation of prostate cancer cells 40. *PTN* encoded Pleiotrophin, which is a secreted cell signaling cytokine that acts as a growth factor associated with the extracellular matrix. Increased serum PTN levels were associated with a high risk of metastasis compared to benign and low risk of metastasis. A high level of tissue PTN was independently index for biochemical recurrence and metastatic progression in early stage 41. *UAP1* encodes UDP-N-acetylglucosamine pyrophosphorylase 1, which was highly over-expressed in prostate cancer and protected cancer cells from endoplasmic reticulum stress 42. Our study first reported the association between BPH and *SCN7A*. In summary, as we can find, the BPH-specific genes screened by our study have also played various roles in normal and abnormal prostate diseases. A more in-depth investigation should be performed to further differentiate BPH from prostate cancer based on these screened characteristic genes.

Besides the expression profile of characteristic genes, we also demonstrate the profile of immune cell infiltration. Chronic inflammation has been suggested as key factor for BPH. Thus, immune cell infiltration can be another good feature variable for differentiating BPH, PC, and normal cases. One review has summarized that histology of all BPH showed inflammatory infiltrates. They introduced various immune cell types and their potential roles in BPH 43. Our study has tried to demonstrate the phenotype of immune cell infiltration. The difference analysis of 28 kinds of immune cell infiltration was performed among BPH, PC, and Normal groups. In contrast to PC, the BPH group showed that the infiltration degree of 17 kinds of immune cells in the BPH group was significantly higher than that in the PC group, while only Gamma delta T cell and Neutrophil were decreased. In contrast to the normal group, the BPH group showed that the infiltration of 7 immune cells was up-regulated, while the infiltration of another 7 immune cells was down-regulated. Compared with the other two groups, the immune cells in the BPH group have a unique tendency: there are 6 types of up-regulated infiltration of immune cells (Immature B cell, Regulatory T cell, T follicular helper cell, Macrophage, MDSC, and Plasmacytoid dendritic cell) and 1 type of down-regulated infiltration of immune cell (Neutrophil). These results preliminarily screened the potential value of immune cell infiltration in the differential diagnosis of BPH.

Specifically, several studies have tried to explore the microenvironment of BPH in depth. In a study performed on mouse, decreased androgen receptor (AR) signaling in prostate luminal cells upregulated cytokines and chemokines, and impaired epithelial barrier function. The immune cell infiltration into the prostate was enhanced 44. Macrophages regulated AR and CD40/CD40L expression to promote inflammation and proliferation as well as inhibit apoptosis of BPH-1 cells through activation of the MAPK signaling pathway 45. One study analyzed the 50 transurethral prostatic resection specimens, each entailing normal prostate, BPH, and high-grade PC, and evaluated the density and phenotype of the immune cells using IHC methods and immunostaining. The increased density of immune cells in BPH suggested that the initial response to cellular damage was mediated by cell-mediated immunity. The decreased density of immune cells in high-grade PC may reflect immunosuppression 46. In the results of our study, pDC and Neutrophil have been proposed as two most important immune cells. The pDCs can detect pathogen-derived nucleic acids and respond with rapid and massive production of type I interferon. Our study has first proposed pDC as the feature immune cell specific to BPH, which provided a high AUC up to 0.834 for distinguishing BPH from Non-BPH. Similar to pDC, Neutrophil has also showed good performance as the feature immune cell of BPH, which provided a high AUC up to 0.823.

Finally, we compared transcriptional differences between BPH and normal tissues and used bioinformatics to screen for small molecule drugs that could reverse this process of BPH occurrence and development. Currently, commonly used drugs for the treatment of benign prostatic hyperplasia (BPH) in clinical practice include adrenergic receptor blockers, alpha-blockers, and 5-alpha-reductase inhibitors. However, there are fewer reports on drugs that directly inhibit prostate cell proliferation or induce apoptosis. Ursolic acid was found to have therapeutic potential for BPH through *in vivo* and *in vitro* experiments. It induces apoptosis in BPH-1 cells and inhibits their proliferation by causing S-phase arrest. The therapeutic effect of ursolic acid was further verified in a BPH rat model. However, there were also limitations. However, this study also has certain limitations. For example, appropriate serum sample data were not included, which could have provided new applications for these novel biomarkers in non-invasive diagnostics. Additionally, the clinical sample data collected was not extensive enough, and further validation is needed in larger independent cohorts in the future.

## Conclusion

The diagnostic biomarkers, microenvironment characteristics, and therapeutic effect of ursolic acid explored in this study offer new diagnostic and therapeutic strategies for BPH.

## Supplementary Material

Supplementary file S1 and file S2.

## Figures and Tables

**Table 1:** The information of data sets.

ID	Normal	BPH	PC	Platform	Counts
GSE7307	12	7	9	GPL570	28
GSE65343	0	10	0	GPL17692	10
GSE119195	3	5	0	GPL6244	8
GSE132714	4	18	0	GPL16791	22
GSE134051	0	39	164	GPL26898	203
GTEx	100	0	0	Illumina	100
TCGA-PC	52	0	499	Illumina	551
Total	171	79	672		922

**Table 2:** Primers used for qRT-PCR.

Gene	Forward primer (5'-3')	Reverse primer (5'-3')
*ALCAM*	TCCTGCCGTCTGCTCTTCT	TTCTGAGGTACGTCAAGTCGG
*COL6A2*	GACTCCACCGAGATCGACCA	CTTGTAGCACTCTCCGTAGGC
*CRISP2*	GGAGCAGAGAGGTAACAACGA	TTGTACTGGTTTTGCGGTCCT
*FOXF2*	AATGCCACTCGCCCTACAC	CGTTCTGGTGCAAGTAGCTCT
*IGF1*	GCTCTTCAGTTCGTGTGTGGA	GCCTCCTTAGATCACAGCTCC
*PTN*	GGAGCTGAGTGCAAGCAAAC	CTCGCTTCAGACTTCCAGTTC
*SCN7A*	CAATGCGGCTTCCATCTTGTG	ACGCAATCAATCAGGACACTAAT
*UAP1*	CTCCAGGCCATGAACTTTGAG	TCCATTCGTGCATCCACATTC
*GAPDH*	CAGCCTCAAGATCATCAGCA	TGTGGTCATGAGTCCTTCCA

**Table 3:** The AUC value of the ROC curve and the optimal cutoff expression value of 8 screened genes.

Gene	BPH vs Normal		BPH vs Cancer		BPH vs Non-BPH	
	AUC Value	Best cut-off expression (Specificity, Sensitivity)	AUC Value	Best cut-off expression (Specificity, Sensitivity)	AUC Value	Best cut-off expression (Specificity, Sensitivity)
*ALCAM*	0.758	5.465 (0.835, 0.569)	0.912	5.603 (0.924, 0.784)	0.88	5.603 (0.924, 0.727)
*COL6A2*	0.762	7.873 (0.797, 0.620)	0.901	7.877 (0.797, 0.862)	0.872	7.877 (0.797, 0.813)
*CRISP2*	0.955	1.358 (0.873, 0.971)	0.922	1.356 (0.873, 0.936)	0.929	1.356 (0.873, 0.943)
*FOXF2*	0.644	3.621 (0.734, 0.538)	0.805	3.552 (0.759, 0.775)	0.772	3.552 (0.759, 0.721)
*IGF1*	0.863	3.913 (0.785, 0.807)	0.899	3.299 (0.949, 0.732)	0.892	3.567 (0.861, 0.784)
*PTN*	0.665	6.733 (0.772, 0.497)	0.851	6.485 (0.835, 0.753)	0.813	6.489 (0.835, 0.688)
*SCN7A*	0.752	3.366 (0.810, 0.573)	0.913	3.135 (0.924, 0.786)	0.881	3.135 (0.924, 0.715)
*UAP1*	0.785	5.612 (0.861, 0.626)	0.953	5.794 (0.949, 0.868)	0.919	5.794 (0.949, 0.784)

## Abbreviations

BPH: Benign Prostatic Hyperplasia
PC: Prostate Cancer
PSA: Prostate Specific Antigen
GEO: Gene Expression Omnibus
GTEx: Genotype-Tissue Expression
TCGA: The Cancer Genome Atlas
DEGs: Differentially Expressed Genes
NES: Normalized Enrichment Score
WGCNA: Weighted Gene Co-Expression Network Analysis
SVM-RFE: Support Vector Machine-Recursive Feature Elimination
LASSO: Least Absolute Shrinkage and Selector Operation
